# Transthyretin-related hereditary amyloidosis with recurrent vomiting and renal insufficiency as the initial presentation: A case report

**DOI:** 10.1097/MD.0000000000005737

**Published:** 2017-03-10

**Authors:** Jing Xu, Meng Yang, Xiaoxia Pan, Xialian Yu, Jingyuan Xie, Hong Ren, Xiao Li, Nan Chen

**Affiliations:** Institute of Nephrology, Department of Nephrology, Ruijin Hospital, Shanghai Jiao Tong University School of Medicine, Shanghai, PR China.

**Keywords:** case report, p.Leu75Pro, renal insufficiency, rs121918079, transthyretin-related hereditary amyloidosis, *TTR* mutation

## Abstract

**Rationale:**

Hereditary amyloidosis is diagnosed worldwidely with an increasing incidence. As the most common form, transthyretin-related hereditary amyloidosis (ATTR amyloidosis) is an autosomal dominant inherited disease due to mutations of TTR. Over the past several decades, more than 130 mutations have been reported. Previous studies suggested that ATTR amyloidosis initially showed polyneuropathy and autonomic dysfunction but later involving many visceral organs, such as kidney.

**Patient concerns:**

A young proband carrying TTR p.Leu75Pro mutation, a reported aggressive variant, initially presenting repeat vomiting and impaired renal function was described in a Chinese family.

**Diagnoses:**

ATTR amyloidosis patient was diagnosed by renal biopsy and gene sequencing.

**Interventions:**

Allograft liver transplantation (LT).

**Outcomes:**

Symptom relief but serum creatinine increased.

**Lessons subsections:**

This case illustrated the clinical and pathologic phenotype of an ATTR amyloidosis patient who initially presented impaired renal function and p.Leu75Pro variant was found by sequencing the coding region of TTR gene. Kidney is one of the most common and vulnerable organs of amyloidosis, and renal function should be closely monitored.

## 1 Introduction

Systemic amyloidosis is a multiorgan disorder characterized by deposition of insoluble, toxic, fibrillary protein aggregates in the extracellular matrix.^[1]^ Hereditary amyloidosis is an autosomal dominantly inherited disease that is associated with genetic variants of various genes, including transthyretin (*TTR*), fibrinogen A α-chain (*Fib*), apolipoprotein AI (*ApoAI*), apolipoprotein AII (*ApoAII*), lysozyme (*Lys*), cystatin C (*Cys*), gelsolin (*Gel*), and beta-2-microglobulin (*β2-MG*). According to the human mutation database,^[2]^ mutated *TTR* is the most frequent cause accounting for 68%. *TTR* is encoded by a single gene on chromosome 18 and more than 130 amyloidogenic *TTR* mutations have been described. Ninety-nine percent of the mutations located at exon 2 to 4, and substitution of methionine for valine at position 30 (Val30Met) is the most common and geographically most widely disseminated.

Transthyretin-related hereditary amyloidosis (ATTR amyloidosis), also known as familial amyloidotic polyneuropathy (FAP), is the most frequent cause in hereditary amyloidosis since it was first identified and described by Portuguese neurologistMário Corino da Costa Andrade in 1952.^[3]^ The age at onset is variable, from 17 to 78 years.^[4]^ ATTR amyloidosis is characterized by a slowly progressive peripheral sensorymotor neuropathy and autonomic neuropathy as well as non-neuropathic changes of cardiomyopathy, nephropathy, vitreous opacities, and central nervous system (CNS) amyloidosis.^[5]^ TTR renal amyloidosis is characterized by early interstitial amyloid deposition in the medulla, even in the absence of clinical nephropathy. Besides, clinical manifestations of patients correlated with glomerular and vascular involvement.^[6]^ Hereditary amyloidosis is progressive, high disabling, and chronic renal failure is a common cause of death. Progressive renal impairment needs more attention.

Few *TTR* mutations have been reported in Chinese patients and none of them represented renal function insufficiency as the first symptom. In our case, we reported a clinical phenotype of TTR amyloidosis caused by p.Leu75Pro mutation in a Chinese family with the proband representing renal function insufficiency at onset. Kidney is the most common and easily affected organ of amyloidosis that should raise more attention of nephrologists.

## 2 Case report

**Figure 1:**
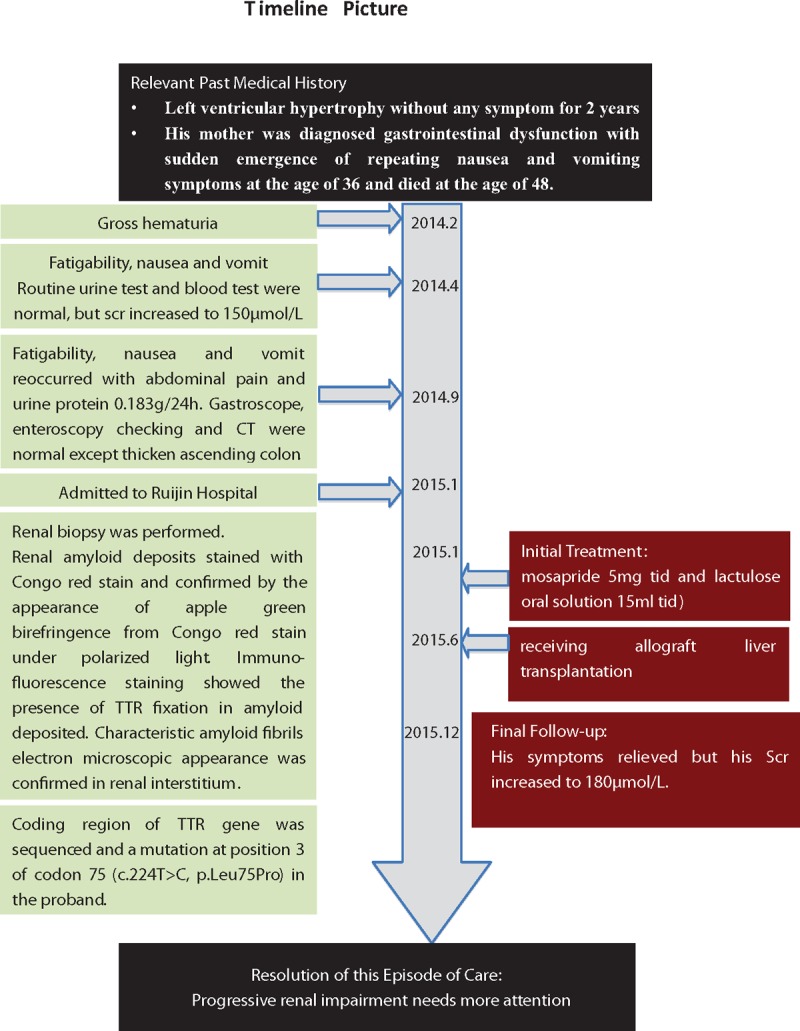
Timeline.

A 26-year-old Chinese male was admitted to our hospital with recurrent vomiting and elevated serum creatinine (Scr) lasting 9 months without any clear inducement factors (Fig. 1).

He was examined to have left ventricular hypertrophy in a physical examination 2 years ago. Due to no symptom, he did not go to hospital for further treatment. Eleven months ago, he went to hospital because of gross hematuria. After 2 months, he felt fatigability, nausea, and vomited several times suddenly. He had normal blood pressure (BP), temperature, regular heartbeat, and rhythm. Physical examination revealed normal vital signs. No abnormity was observed in routine urine test and blood test, but blood biochemical test revealed his Scr increased to 150 μmol/L (Chronic Kidney Disease Epidemiology Collaboration [CKD-EPI] equation estimated glomerular filtration rate [eGFR]: 55 mL/min/1.73 m^2^). After receiving proper treatment, fatigability, nausea, and vomit were obviously relieved. But 5 months later, these symptoms reoccurred and he felt abdominal pain with urine protein 0.183 g/24 h (urinary volume: 1.8 L). Gastroscope and enteroscopy checking were normal. Besides, no abnormality was detected by abdomen computed tomography (CT) except thicken ascending colon. The echocardiography showed thickening of the left ventricular wall and normal wall motion, suggesting nonobstructive hypertrophic cardiomyopathy. Moreover, cardiac magnetic resonance image (MRI) revealed left ventricular hypertrophy, suggesting hypertrophic cardiomyopathy. Over the past 9 months, he lost weight obviously, BMI 17.58 kg/m^2^ (height: 171 cm, weight: 51.4 kg), occasionally with blurred vision and conjunctival congestions. In addition, his mother had similar medical history. His mother was diagnosed gastrointestinal dysfunction with sudden emergence of repeating nausea and vomiting symptoms at the age of 36. After 12 years’ treatment, she had no remission and felt depressed. Finally, she died at the age of 48.

No obvious abnormality was found in examination findings on admission: BP 98/60 mm Hg, temperature 37°C, heart rate (HR) 85 bpm with order rhythm, except systolic rumor between the third and fourth rib of left margin of heart. He had no edema and any discomfort in abdomen and nervous system. The electrocardiogram revealed sinus rhythm with extreme clockwise translocation.

No abnormality was observed in routine test, such as urine, blood, and stool. And the specific gravity of urine and urine pH was within normal limits. Quantification of proteinuria showed 0.126 g/24 h (urinary volume: 1.8 L). Tests of renal function showed Scr136 μmol/L (CKD-EPI eGFR: 61 mL/min/1.73 m^2^), urea nitrogen 7.9 mmol/L, uric acid 449 μmol/L. Hemoglobin was 11.3 g/dL, leucocyte count 7.9∗10^9^/L (normal differential), and platelets 173∗10^9^/L. Serum electrolyte analysis showed sodium 138 mmol/L, potassium 3.75 mmol/L, serum carbon dioxide 31.7 mmol/L, calcium 2.29 mmol/L, and phosphate 1.24 mmol/L. Liver function laboratory tests were normal, with alanine aminotransferase 22 IU/L, aspartate transaminase 23 IU/L, alkaline phosphatase 56 IU/L, and albumin 42 g/L. Blood lipid, glucose, thyroid function, and myocardial enzyme were normal. ANA, ENA, anti-GBM, and ANCA were negative. ANCA were tested by enzyme-linked immunosorbent assays for anti-PR-3 and anti-MPO antibodies. Immunoglobin (Ig) A 164 mg/dL, IgG 1270 mg/dL, IgM 194 mg/dL, C3 was 63 mg/dL, and C4 17 mg/dL. Urine and serum protein immunofixation electrophoresis were both normal, with IgA, G, M, light chain κ, and λ all negative. Serologic tests for hepatitis B virus, hepatitis C virus, and HIV were negative. Tumor markers such as AFP, carcinoembryonic antigen, CA 199, and CA 125 were all negative. Moreover, urine ACR was less than 2.5 mg/mol and 24 h urine electrolyte excretion (urinary volume: 1.3 L) was decreased with sodium 37.7 mmol/24 h, potassium 13.13 mmol/24 h, chloridion 39 mmol/24 h, calcium 0.42 mmol/24 h, and phosphate 3.35 mmol/24 h. In addition, bone marrow aspiration was performed and no abnormal plasmocyte was found, which ruled myeloma out.

**Figure 2:**
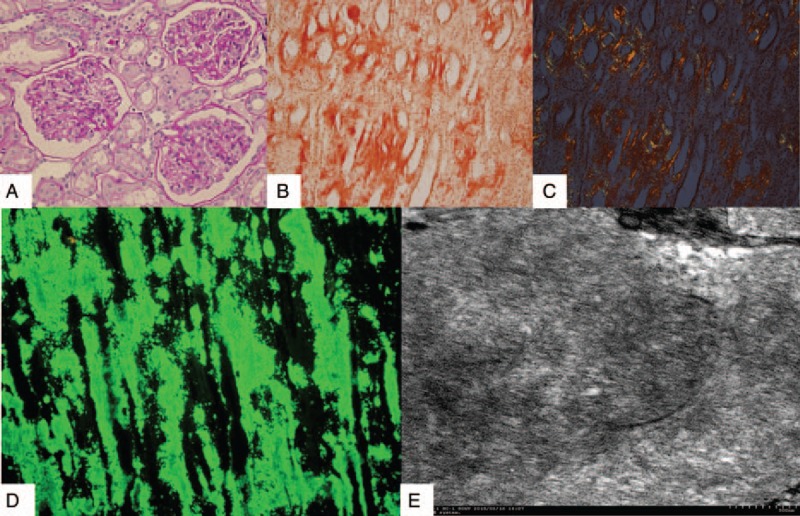
Renal biopsy results (A) PAS stain (×400). (B) Congo red stain (×200). (C) Congo red stain under polarized light (×200). (D) Immunofluorescence stain of TTR (×200). (E) Characteristic amyloid fibrils in electron microscopy (×90,000). PAS = periodic acid-Schiff stain.

Ultrasound revealed normal kidneys, with left kidney 98∗41 mm and right kidney 100∗32 mm. To investigate the reason of the elevated serum creatinine, a renal biopsy was performed on January 13, 2015. The biopsy showed global sclerosis in 9 of 28 glomeruli. For light microscopy (LM), the serial sections were stained with H&E, periodic acid-Schiff, Masson's trichrome and Jones’ methenamine silver stains, and Congo red. LM (Fig. 2A periodic acid-Schiff stain [PAS] staining) showed that most capillary loops were open and expansion of individual mesangium by a few weak PAS-positive, homogeneous, and amorphous substances without proliferation of mesangial cells. Renal amyloid deposits stained with Congo red stain and confirmed by the appearance of apple green birefringence from Congo red stain under polarized light (Fig. 2B, C). They predominated in interstitial tissue, and mainly in renal medulla. Immunofluorescence staining was negative, including IgA, IgG, IgM, C3, C4, C1q, and fibrinogen. Immunoreactivity of renal tissue AA amyloid deposition and light chain κ or λ were all negative. But immunofluorescence staining showed the presence of TTR fixation in amyloid deposited (Fig. 2D). Characteristic amyloid fibers in renal intertsitium were confirmed by tranmission electron microscopy (Fig. 2E). The renal biopsy was jointly reviewed by 2 pathologists (XP and JX).

**Figure 3:**
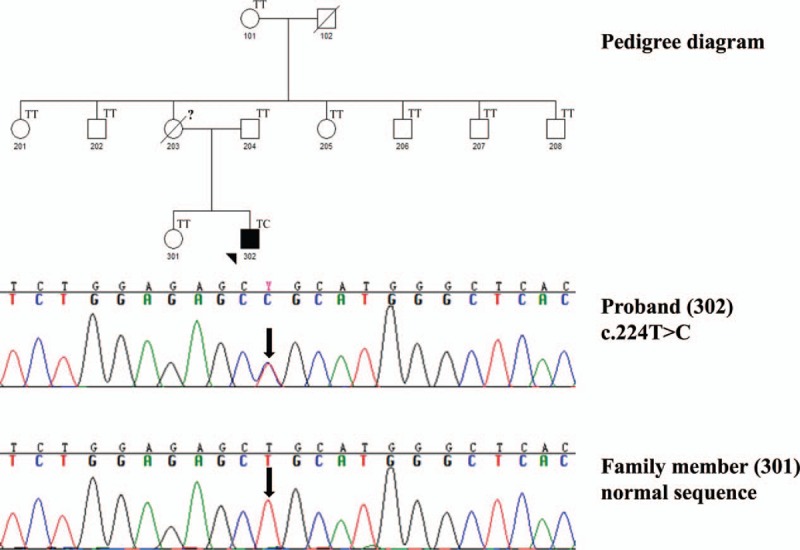
Pedigree diagram and sequence results.

After assessing the family history over 3 generations, genomic DNA of all family members (except his mother) was extracted from peripheral blood by using the standard phenol-chloroform extraction method. Polymerase chain reaction was used to amplify the coding region of *TTR* gene (the oligonucleotide primers in Table 1) and *TTR* mutations were screened by direct sequencing using an ABI Prism 3100 Genetic Analyzer (Applied Biosystems). The sample sequences were compared with the genomic DNA sequence of *TTR* [Ensembl: ENSG00000118271; Assembly: GRCh38.p2 (GCF_000001405.28)]. Direct DNA sequencing of all family members’ *TTR* gene revealed a mutation at position 3 of codon 75 (c.224T>C, p.Leu75Pro) (previously reported as p.Leu55Pro) in the proband (Fig. 3).

**Table 1:**
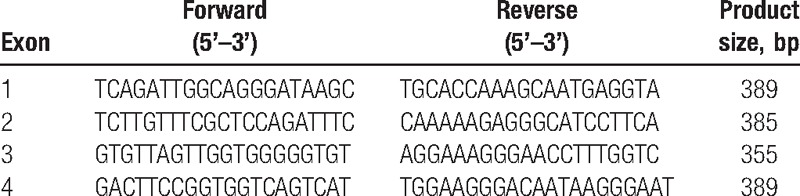
Sequence of *TTR* gene oligonucleotide primers.

Finally, combining clinical, laboratory examination, renal biopsy, and gene diagnosis, this patient was diagnosed as ATTR amyloidosis that was caused by *TTR* p.Leu75Pro mutation. To relieve gastrointestinal symptoms, he was treated by mosapride 5 mg three times a day (tid) and lactulose oral solution 15 mL tid. After 5 months, he received allograft liver transplantation (LT). Now he felt symptom relief, but his Scr increased to 180 μmol/L.

This study was approved by the Institutional Review Board of Ruijin Hospital, Shanghai Jiao Tong University School of Medicine. It was in accordance with the principle of the Helsinki Declaration II. The written informed consent was obtained from each participant.

## 3 Discussion

This is the first report on ATTR amyloidosis that was caused by the *TTR* p.Leu75Pro (rs121918079) mutation, legacy name p.Leu55Pro, in mainland China. Compared with previous reports of *TTR* p.Leu75Pro mutation,^[7–9]^ the young proband initially presented gastrointestinal symptoms, impaired renal function, and lacked neurological symptoms. Then, he came to nephrology department to figure out the reason of his elevated serum creatinine, he received biopsy, and the pathological results suggested hereditary amyloidosis that was validated by genomic sequence.

TTR is a tetramer that acts as hormone transporter for thyroid hormone and retinol-binding protein. Mutated transthyretin (TTR) is the most frequent cause in hereditary amyloidosis. p.Leu75Prois a rare mutation and it was firstly reported in 1992 by Jacobson et al.^[7]^ He found that the mutation might be an aggressive variant in ATTR amyloidosis and be responsible for patients’ early onset, aggressive, widespread amyloid deposition with cardiac and neurologic involvement. In 1994, Yamamoto et al^[8]^ reported another FAP family with *TTR* p.Leu75Pro, showing an early-onset and a fatal outcome before age 30. The *TTR*p.Leu75Pro was considered to be more serious than other forms of ATTR amyloidosis. TTR p.Leu75Pro was an unstable amyloidogenic protein than the wild-type and it easily broke down to monomers. At the earlier stage, partially unfolded monomer and subsequent aggregates presented.^[10]^ Furthermore, a zinc-dependent pathway might be involved in the production of early amyloidogenic intermediates of p.Leu75Pro transthyretin amyloid fibrils.^[11]^

ATTR amyloidosis related renal failure (eGFR<60 mL/min/m^2^) was reported to affect about 37% patients of FAP type I, which was caused by the most common mutation (*TTR* Val30Met).^[12]^ And end-stage renal disease (ESRD) patients with *TTR*Val30Met mutation started dialysis at a mean age 51.5 years.^[13]^ Besides, previous study revealed that screening of microalbuminuria might be an important predictor to assess ATTR amyloidosis onset and predict overt nephropathy as a noninvasive examination.^[14]^ Few studies of long-term prognosis of p.Leu75Pro were reported. This remains much work to be done by cooperation of doctors from different departments including nephrologists. In summary, kidney involvement was vital and should not be overlooked.

Hereditary amyloidosisis was thought to be extremely rare and the clinical phenotype was nonspecific. So, light-chain (AL) amyloidosis was often misdiagnosed by clinical and laboratory findings with absence of family history in the past. By studying 350 patients who were diagnosed AL amyloidosis, Lachmann et al^[15]^ found amyloidogenic mutations were present in 34 of the 350 patients (9.7%), most often in the genes encoding fibrinogen A a-chain (18 patients) and transthyretin (13 patients). Besides, the diagnosis of hereditary amyloidosis was confirmed by additional investigations in all 34 of these patients. This study suggested that hereditary amyloidosis was grossly neglected. Same situation occurred in hereditary renal amyloid,^[16]^ which should raise the attention of nephrologists. Besides clinical, laboratory examination, and renal biopsy, genotyping is an extremely important method to avoid misdiagnosis. Seeking the genetic cause is recommended in all patients with amyloidosis that is not the reactive systemic amyloid A type and in whom confirmation of the AL type cannot be obtained.^[15]^

So far, there is no optimal treatment for ATTR amyloidosis and LT may be a good choice. TTR is predominantly synthesized by liver, so LT seems to be effective in halting amyloid deposition derived from a *TTR* variant in serum and may halt the progression of kidney damage caused by amyloid deposition.^[17]^ Kon et al^[9]^ reported the 1-year outcome for an ATTR amyloidosis patient carrying p.Leu75Pro mutation. He was treated with LT and tafamidis and no progression of the disease was observed, indicating that combination therapy may be beneficial for patients with this aggressive mutation.^[9]^ But the prognosis after operation might be closely related to the severity of amyloid deposits in renal glomeruli.^[18]^ Although renal biopsy is not routinely required, it does make sense and might be recommended when determining the indications and contraindications for LT in ATTR amyloidosis patients.

## 4 Conclusion

This case illustrated the clinical and pathologic phenotype of an ATTR amyloidosis patient in a Chinese family. The coding region of *TTR* gene was sequenced and a mutation at position 3 of codon 75 (c.224T>C) p.Leu75Pro, legacy name p.Leu55Pro, was found. When patients come to nephrology department due to elevated Scr levels with unknown causes, she/he is under a high suspicion of hereditary amyloidosis when combined with multiorgan involved symptom. Especially when other family members have similar medical history or symptom that do not relieve after lengthy treatment, nephrologists should consider hereditary amyloidosis related renal dysfunction. In addition, renal biopsy and gene sequencing are important auxiliary diagnoses for hereditary amyloidosis and for avoiding misdiagnosing AL amyloidosis. Kidney is one of the most common and vulnerable organs of amyloidosis, and renal function should be closely monitored.
